# Serum Appetite-Regulating Hormone Levels in Cystic Fibrosis Patients: Influence of the Disease Severity and the Type of Bacterial Infection—A Pilot Study

**DOI:** 10.3390/nu15081851

**Published:** 2023-04-12

**Authors:** Sabina Galiniak, Rafał Podgórski, Marta Rachel, Artur Mazur

**Affiliations:** 1 Institute of Medical Sciences, Medical College, Rzeszow University, Warzywna 1a, 35-310 Rzeszow, Poland; 2 State Hospital 2 in Rzeszów, Lwowska 60, 35-301 Rzeszów, Poland

**Keywords:** cystic fibrosis, appetite, hormones, endocrine system

## Abstract

Cystic fibrosis (CF) belongs to the most common inherited diseases. The severity of the disease and chronic bacterial infections are associated with a lower body index, undernutrition, higher number of pulmonary exacerbations, more hospital admissions, and increased mortality. The aim of our study was to determine the impact of the severity of the disease and the type of bacterial infection in 38 CF patients on the serum level of appetite-regulating hormones including leptin, ghrelin, neuropeptide Y, agouti-signaling protein, proopiomelanocortin, kisspeptin, putative protein Y, and α-melanocyte-stimulating hormone. The patients were divided according to the severity of the disease according to spirometry and the type of chronic bacterial infection. We found that leptin level was significantly higher in patients with severe CF than in patients with mild disease (20.02 ± 8.09 vs. 12.38 ± 6.03 ng/mL, *p* = 0.028). Furthermore, leptin level was elevated in patients with chronic infection with *Pseudomonas aeruginosa* compared to uninfected participants (15.74 ± 7.02 vs. 9.28 ± 1.72 ng/mL, *p* = 0.043). The severity of the disease and the type of bacterial infection did not affect the levels of other appetite-regulating hormones. Moreover, we found a positive correlation between pro-inflammatory interleukin-6 and leptin level (*p* = 0.0426, R = 0.333). Taken together, our results indicate that both the severity of the disease and the type of bacterial infection are associated with elevated leptin levels in CF patients. Future CF treatment strategies should consider possible disturbances in the hormones that regulate appetite and the factors that influence their levels.

## 1. Introduction

Cystic fibrosis (CF) is an autosomal recessive genetic disease characterized by chronic obstructive lung disease and caused by mutations in the CF transmembrane conductance regulator (CFTR) gene. The organs most affected are the respiratory tract, which presents recurrent infections, and the gastrointestinal system, with clinical manifestations, that include malabsorption, which leads to growth retardation caused by progressive pancreatic insufficiency. Due to the fact that many patients with CF develop progressive lung disease, spirometry measurements, in particular forced expiratory volume (FEV_1_), are used as markers of disease severity and the prediction of survival [1,2]. FEV_1_ decline in CF is correlated with the lower body mass index (BMI), *Pseudomonas aeruginosa* infection, pancreatic insufficiency, CF-related diabetes, and intensification of oxidative stress [3,4,5]. Although CF is a systemic disease, the most severe symptoms are related to the lungs; therefore, *P. aeruginosa*, *Staphylococcus aureus*, and *Burkholderia cepacia* are the most important infectious agents in patients with CF [6,7]. *P. aeruginosa* remains the most common bacterial pathogen, detected in approximately 50% of CF patients in general and in approximately 80% of adults [7]. Infection with *P. aeruginosa* is associated with undernutrition, a higher number of pulmonary exacerbations, more hospital admissions, and increased mortality [8,9,10,11]. Lung disease, *P. aeruginosa*, and poor nutritional status are the three keystones of the clinical symptoms [12]. There are few reports in the literature that describe the impact of the severity of the disease and bacterial infections on the level of appetite-regulating hormones. Data mainly concern ghrelin and leptin, whose levels are disrupted as lung function declines [13,14,15]. In addition to ghrelin and leptin, several other hormones are involved in the regulation of appetite. Neuropeptide Y (NPY) is one of the most potent orexigenic peptides found in the brain, which stimulates food intake with a preferential effect on carbohydrate intake [16]. Agouti-signaling protein (ASP) and α-melanocyte-stimulating hormone (α-MSH) are involved in skin and hair pigmentation, but they are also associated with eating behavior, energy homeostasis, and adipose tissue deposition [17,18,19]. Proopiomelanocortin (POMC) is transformed in a tissue-specific manner to produce biologically active peptides, among others, such as ACTH, α-MSH, or the opioid peptide β-endorphin that are expressed primarily in the pituitary and brain [20]. POMC-derived peptides may have the opposite effect on appetite regulation [21,22]. Moreover, kisspeptin (KISS) is adipokine which is involved in energy metabolism, development of gonads, trophoblast invasion, pregnancy, lactation, and homeostasis. KISS is also expressed and regulated in many extrahypothalamic tissues, such as the pancreas, liver, placenta, and adipose tissue [23]. Putative protein Y (PYY) is a gut hormone that inhibits food intake, stomach motility, and intensifies the absorption of water and electrolytes in the colon [24]. Therefore, the aim of our study was to estimate the impact of the severity of the disease and the type of bacterial infection in CF patients on the serum level of appetite-regulating hormones including leptin, ghrelin, NPY, ASP, POMC, KISS, PYY, and α-MSH.

## 2. Materials and Methods

### 2.1. Ethical Issues

The Bioethics Committee of Rzeszów University approved the study design (2022/023). The study was carried out in accordance with Good Clinical Practice and the Declaration of Helsinki. Informed consent was obtained from each participant and/or their parent(s) or legal guardian(s) at the beginning of the study.

### 2.2. Study Design and Participants

Thirty-eight non-Hispanic white patients with CF and sixteen control subjects were included in this single-center cross-sectional study. Recruitment for the study took place at the local CF care center at the Provincial Hospital No. 2, St. Queen Jadwiga, in Rzeszow. The study was carried out between February 2021 and October 2021.

The diagnosis of CF was confirmed by the standard measurement of sweat chloride and newborn bloodspot screening. The CF gene mutation test was used to identify mutations in the CFTR gene. Other criteria for inclusion in the study were FEV_1_ greater than 35% and no hospitalizations within 30 days of screening. Exclusion criteria were prior history of any type of diabetes or organ transplantation, smoking within the last two months, pregnancy, mental and cardiac diseases, and liver failure and recent pulmonary exacerbation for 2 months prior the study. In addition, participants who could not perform a spirometry test or did not sign a written consent were also excluded. All patients had pancreatic insufficiency which was defined as having a low fecal elastase level (<200 μg/g stool).

All CF patients received treatment as recommended (Creon 25000, Solvay Pharmaceutical Inc., Marietta, GA, USA; DNase I recombinant, Pulmozyme, Genentech Inc., San Francisco, CA, USA; ADEK tablets, Scandipharm, Birmingham, AL, USA; nutrition drinks, Nutrison Protein Plus, Nutricia, Poland; and inhalation of 3–7% sodium chloride). Patients were not treated with CFTR modulators. None of our patients used appetite stimulants.

Healthy participants were recruited among children and adults who came to the local clinic to have check-ups at the same time. Healthy volunteers had no disease in medical history and did not take any drugs during the 4 weeks preceding the study. BMI was calculated as kg/m^2^ for all participants > 20 years or Z-score for younger volunteers.

### 2.3. Spirometry

All study volunteers had a spirometry test using a standard device (Lungtest 1000, MES, Kraków, Poland) in accordance with the guidelines of the American Thoracic Society [25]. The maximum of three appropriate measurements is recorded with <15% variation. The mean value of the last half year for FEV_1_ expressed as a percentage of the predicted value for age and sex was calculated. CF subjects were divided into three groups according to the disease severity based on FEV_1_ results—mild disease (FEV_1_ > 75% predicted), moderate disease (FEV_1_ from 45% to 75% predicted), and severe disease (FEV_1_ < 45% predicted) [3,13].

### 2.4. Sputum Collection

Hypertonic saline was used for sputum induction and deep throat swab specimens were cultured for bacterial pathogens. Bacterial and fungal infections were classified according to the criteria [26]. Antimicrobial drug susceptibility tests were performed according to the recommended methodology [27]. Chronic colonization by *P. aeruginosa* was defined by at least 3 positive sputum tests for that strain or the continuous presence of these bacteria over 12 months before to the study. Based on the results of microbiological tests, patients were divided into four groups according to bacterial infection—*P. aeruginosa*, *S. aureus*, colonized with *P. aeruginosa* and *S. aureus*, and those who did not demonstrate any bacterial growth on sputum cultures. The duration of infection was the time difference (in years) between the initial and final positive culture.

### 2.5. Blood Sampling

Blood samples were taken from each CF patient and healthy volunteer after an overnight fast, centrifuged (1500× *g*, 10 min, 4 °C), aliquoted, and frozen at −80 °C in cryovials according to standard procedure. The collected serum samples were not stored for more than 3 months and were thawed only once on the day of analysis.

### 2.6. Blood Counts and Serum Analysis

The complete blood count was performed by using a standard hematology analyzer (Siemens Healthineers, Erlangen, Germany). Interleukin 6 (IL-6) was estimated using commercially available enzyme-linked immunosorbent assays (ELISA) (R&D Systems, Minneapolis, MN, USA) in line with the manufacturer’s instructions.

### 2.7. Appetite-Regulating Hormones Determination

Serum levels of leptin, ghrelin, NPY, ASP, POMC, KISS, PYY, and α-MSH were determined in duplicates with previous dilution using commercially available the ELISA kits (Wuhan Fine Biotech Co., Ltd., Wuhan, China) according to the manufacturers’ instructions. The limits of detection for hormones were as follows: leptin: 18.75 pg/mL; ghrelin: 1.125 pg/mL; NPY: 9.375 pg/mL; ASP: 0.938 pg/mL; POMC: 0.094 ng/mL; KISS: 0.094 ng/mL; PYY: 3.75 pg/mL; and α-MSH: 7.5 pg/mL. The intra-assay coefficients of variation were lower than 8%, and the inter-assay coefficients of variation were lower than 10% for each ELISA.

### 2.8. Statistical Analyses

All Statistical analyses were conducted using statistical packages STATISTICA (version 13.3, StatSoft Inc., 2017, Tulsa, OK, USA). Data were presented as mean and SD, as well as range. Variables did not follow a normal distribution, which was validated using the Shapiro–Wilk test and skewness due to the non-parametric tests that were applied. The Mann–Whitney U test or the Kruskal–Wallis–ANOVA were used. Spearman’s rank correlation coefficient analysis was used to estimate the correlation between IL-6 and leptin level, assuming linear dependence. A *p* > 0.05 was assumed to be statistically significant.

## 3. Results

Our study cohort consists of seventeen females and eleven males in the CF patient group. Ten females and six males were included in the control group. Basic clinical data of the study participants are shown in Table 1.

There was no difference in the age of the study groups (19.6 ± 7.9 vs. 19.3 ± 7.3 years old). CF patients had similar BMI to healthy controls. Among patients with CF, 30 participants were homozygous for ΔF508, while 8 were heterozygous. CF participants had significantly higher white blood cell count. Lung function, determined by spirometry, was worse in the CF group than in healthy controls. Based on the results of spirometry, we divided the CF participants into three groups depending on the severity of the disease. More than 60% of the patients had mild disease, almost 24% of the patients had moderate disease, while the rest of the patients had severe disease as FEV_1_ was below 45%. Moreover, 9 participants with CF were colonized with *P. aeruginosa*, 11 were infected with *S. aureus*, 9 were co-infected with *P. aeruginosa* and *S. aureus*, while 9 did not demonstrate any bacterial growth on sputum cultures. The average duration of *P. aeruginosa* infection was 13.63 years, while *S. aureus* infection was 10.45 years. The average duration of co-infection with *P. aeruginosa* and *S. aureus* was 12.2 years. In our study, there was no statistical difference in the age of patients with mild, moderate, and severe CF (mean age: 17.3 ± 6.8 vs. 21.5 ± 6.5 vs. 24.8 ± 8.2, respectively, *p* = 0.058). Regarding the age of the patients and the type of bacterial infection, uninfected patients were younger than *P. aeruginosa* patients (*p* = 0.03). However, there was no statistical difference between the other study groups (*P. aeruginosa* group: 23.3 ± 5.5 years, *S. aureus* group: 19 ± 4.3, coinfected with *P. aeruginosa* and *S. aures* group: 21.1 ± 11.1 years, uninfected group: 14.6 ± 5 years).

**Figure 1:**
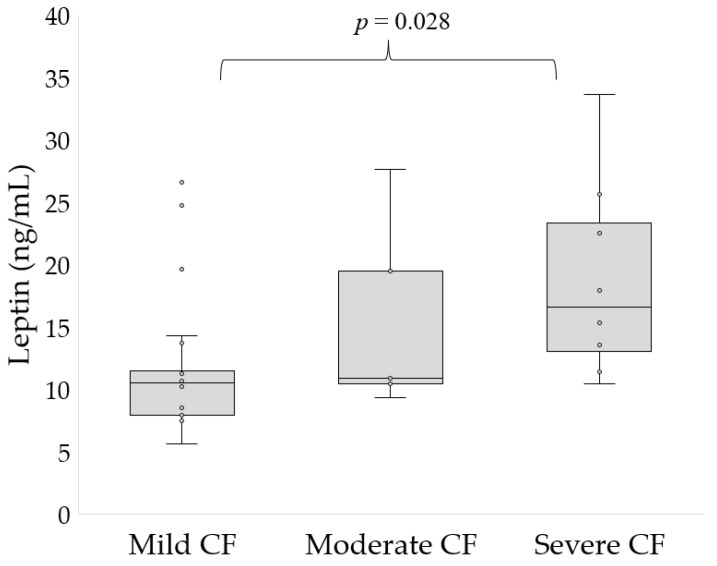
Levels of leptin depending on the disease severity.

We found that leptin level was significantly increased in participants with severe CF compared to those with mild disease (20 ± 8.1 vs. 12.4 ± 6 ng/mL, *p* = 0.028, Figure 1). There were no differences in patients with moderate CF and mild CF or severe CF. The level of leptin in healthy subjects was 6.5 ± 2.6 ng/mL. As the severity of the disease increased, we observed an increase in leptin levels.

Table 2 presents the level of appetite regulating hormones according to the severity of the disease. The degree of disease severity did not affect the level of other tested hormones. The level of other hormones in healthy subjects was as follows: ghrelin—979.8 ± 285 pg/mL; ASP—16.9 ± 4.1 pg/mL; NPY—295.5 ± 48.2 pg/mL; POMC—14.7 ± 6.2 ng/mL; KISS—2.3 ± 0.6 ng/mL; PYY—73.9 ± 16.8 pg/mL; and α-MSH—14.5 ± 1.8 pg/mL.

**Figure 2:**
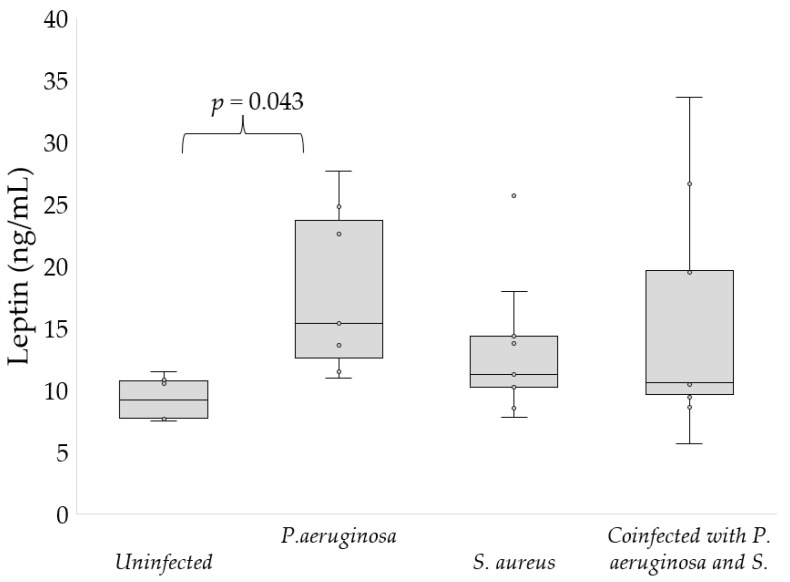
Levels of leptin depending on the type of bacterial infection.

Regarding bacterial infection, leptin level was significantly higher in patients infected with *P. aeruginosa* than in uninfected participants (15.7 ± 7 vs. 9.3 ± 1.7 ng/mL, *p* = 0.043, Figure 2). Infection with *S. aureus* and co-infection with *P. aeruginosa* and *S. aureus* had no influence on the level of leptin.

Table 3 shows the level of appetite regulating hormones according to the type of bacterial infection. Besides leptin, we found no difference in hormone levels depending on the bacterial infection.

**Figure 3:**
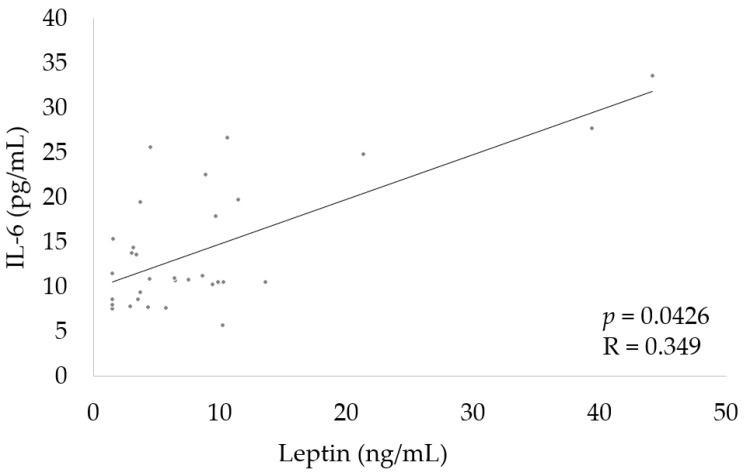
Correlation between IL-6 and leptin.

Finally, we determined the correlation between leptin and IL-6. We observed a positive significant correlation of IL-6 with leptin (Figure 3).

## 4. Discussion

The main findings of this study include the impact of the severe form of CF and chronic *P. aeruginosa* infection on leptin levels. We showed that both severe disease and chronic *P. aeruginosa* infection were associated with elevated leptin levels. In addition, we found that neither the severity of the disease nor the type of bacterial infection affects the level of other tested hormones that regulate appetite (ghrelin, NPY, ASP, POMC, KISS, PYY, and α-MSH).

Due to the fact that nutritional status is intimately related to lung function and survival outcomes, the prevention of malnutrition is fundamental to the care of patients with CF [28]. Malnutrition in CF is primarily due to the following factors: nutrient malabsorption and fecal energy loss due to pancreatic insufficiency, increase in the energy expenditure related to chronic inflammation, breathing efforts, and loss of appetite [29]. Malnutrition may accelerate the progression of the disease; however, being underweight is known to be associated with deterioration in the lung function and higher morbidity and mortality in patients with CF [30,31]. Additionally, it appears that the low body mass in CF patients may be associated with disturbances in the levels of hormones that regulate appetite [13,14]. Currently, reports on levels of appetite-regulating hormones in CF are inconclusive and suggest that, in addition to the disease itself, many factors, including types of CFTR mutations, lifestyle, and nutrition, may influence hormone levels [13,14,32,33,34,35]. Moreover, hormonal disorders of CF are more common in the elderly and are expected to become more frequent as medical care improves and the population ages [36,37].

Our results contradict the findings presented in the study by Cohen et al., in which plasma leptin levels were decreased in patients with severe CF compared with healthy subjects and those with mild and moderate disease [14]. No correlation between FEV_1_ and leptin level was previously reported in CF patients in the study by Nowak et al. [33]. However, in our previous study, we found a negative correlation between leptin levels and FEV_1_ [13]. High levels of leptin may contribute to the anorexia, poor weight gain, and development of children with CF [38]. Moreover, a higher level of leptin was reported in patients with acute exacerbation of chronic obstructive lung disease than in participants with stable disease and healthy controls [39].

The mechanism of elevated leptin levels is not fully known; however, it could be associated with increased levels of tumor necrosis factor-α as well as IL-1 and -6, as these cytokines mediate the inflammatory response to chronic infection and may induce leptin production [40,41]. Both severe disease and *P. aeruginosa* infection are associated with the enhanced release of pro-inflammatory cytokines and reduced synthesis of anti-inflammatory mediators [41,42]. Therefore, there may be a positive correlation between IL-6 and leptin determined in our study. Similarly, this positive association has also been described in previous reports in patients with obesity-related inflammation [43,44]. Furthermore, a study on cellular models showed that hypoxia intensifies leptin expression through activation of the leptin gene promoter [45,46]. Episodic hypoxia may occur during periods of physiological stress in CF, such as sleep, exercise, and disease exacerbations [47]. A recent study by Polito et al. reported that leptin levels were slightly decreased in sedentary CF patients as compared with active patients, although the differences were not significant, which indicates that lifestyle and level of physical activity also affects the level of this hormone [48].

Moreover, some reports revealed that in the presence of high leptin concentration, the production of reactive oxygen species (ROS) is stimulated by many cell types [49,50]. Leptin is important for the development and regulation of the redox system; hence, increased leptin concentrations may induce the release of ROS and promote inflammation which may be one of the causes of the exaggerated oxidative stress in CF [51,52]. Therefore, a high level of leptin may be related with an increased level of ROS in patients with severe CF and patients with chronic colonization with *P. aeruginosa* [3]. Furthermore, hypoxia can lead to a deterioration in the lung function by upregulating inflammation, promoting the growth of *P. aeruginosa* and inducing human preadipocytes to synthesize and secrete leptin [47,53].

In this study, concentrations of other appetite-regulating hormones were independent of disease severity and type of bacterial infection. Nowak et al. also did not report a correlation between the NPY level and the spirometry results [33]. However, a study on a group of adult CF patients showed that in severe CF patients, ghrelin levels were significantly elevated compared to controls and those with mild and moderate disease, which is inconsistent with our results [14]. On the other hand, the expression of the ghrelin receptor in the CF participants with normal BMIs was similar to that of controls; it lowered during an acute exacerbation related with weight loss and returned to baseline after treatment [54]. It should also be mentioned that in our study, differences in ghrelin levels were close to statistical significance between the groups according to the type of bacterial infection. There are no data in the literature on the severity of the disease and the type of bacterial infections that may affect the levels of other hormones that were analyzed in this study.

Future CF research should consider possible disturbances in the hormones that regulate appetite and other factors that influence hormone levels, such as the type of CFTR mutation, diet, physical activity, or treatment, including therapy with CFTR modulators. The detection of appetite-regulating hormone disorders should be an essential part of high-quality medical care for patients with CF.

Although our study shows for the first time an association between the severity of the disease and the type of bacterial infection on the level of appetite-regulating hormones in CF, some limitations of the study should be mentioned. This was a single-center study with a limited number of patients. Moreover, the number of patients in each group was small. Therefore, it is necessary to be careful when interpreting and comparing the results. Furthermore, we have not investigated other factors that influence hormone levels, such as exercise or steroid hormones. Nevertheless, the study also has strengths. The patient cohort was well characterized clinically. Additionally, this study measured the levels of multiple hormones in the same patients.

## 5. Conclusions

Taken together, our results indicate that both the severity of the disease and the type of bacterial infection are associated with elevated leptin levels in CF patients. In addition, there does not appear to be a relation between the severity of the disease, the type of bacterial infection, and the rest of the hormones that regulate appetite in CF. Future CF treatment strategies should consider possible disturbances in the hormones that regulate appetite and the factors that influence their levels.

## Figures and Tables

**Table 1:** Basic clinical parameters of study participants *.

		CF	Healthy Controls	*p*
Sex (F/M)		17/21	10/6	
Age (years)	mean ± SD	19.6 ± 7.9	19.3 ± 7.3	0.855
	range	10–39	10–38	
BMI (kg/m^2^), patients > 20 years	mean ± SD	21.1 ± 2.9	23.2 ± 2.4	0.068
	range	17.2–25.9	18.7–25.6	
BMI (Z-score), patients < 20 years	mean ± SD	−0.8 ± 1.1	0.2 ± 0.5	0.433
	range	−2.1–1.1	−0.2–1	
Genotype				
Homozygous ΔF508, *n* (%)		30 (78.9)	–	–
Heterozygous ΔF508, *n* (%)		8 (21.1)	–	–
Clinical laboratory markers				
WBC (10^3^/µL)	mean ± SD	10 ± 3.6	7.5 ± 2.3	0.022
	range	5.1–19.3	4.3–10.5	
NEU (%)	mean ± SD	61 ± 15.3	59.1 ± 6.1	0.605
	range	25.1–82.3	50.6–68.6	
Pulmonary function				
FEV_1_ (% predicted)	mean ± SD	86.4 ± 27	102.4 ± 8.2	0.006
	range	35–142	97–127	
Severity of disease				
Mild (FEV_1_ > 75%), *n* (%)		23 (60.5)	–	–
Moderate (FEV_1_ > 45%, <75%), *n* (%)		9 (23.7)	–	–
Severe (FEV_1_ < 45%), *n* (%)		6 (15.8)	–	–
Bacterial infection				
*P. aeruginosa*, *n* (%)		9 (23.7)	–	–
*S. aureus*, *n* (%)		11 (28.9)	–	–
Co–infected with *P. aeruginosa* and *S. aureus*, *n* (%)		9 (23.7)	–	–
Uninfected, *n* (%)		9 (23.7)	16 (100)	–

* BMI: Body mass index; WBC: White blood cells; NEU: Neutrophils; FEV_1_: Forced expiratory volume in 1 s.

**Table 2:** Levels of hormones depending on the disease severity *.

	Mild CF 10 F/13 M	Moderate CF 4 F/5 M	Severe CF 3 F/3 M	*p*
Ghrelin (pg/mL)	687.6 ± 197.4 (345.5–1070.5)	556 ± 274.3 (192.3–828)	616.2 ± 165.5 (419.3–785.1)	0.428
ASP (pg/mL)	14 ± 2.1 (11.7–19.1)	14.1 ± 2.2 (11.9–17.9)	13.6 ± 2.9 (11.9–18.7)	0.199
NPY (pg/mL)	290.1 ± 58 (167.6–381.6)	271.5 ± 53.7 (211.1–376.4)	242.7 ± 37.3 (210.6–300.2)	0.596
POMC (ng/mL)	6.9 ± 4.8 (2–15.5)	5.6 ± 3.1 (1.9–10.9)	3.8 ± 2.4 (2.1–8)	0.558
KISS (ng/mL)	1.8 ± 0.4 (1.1–3)	1.5 ± 0.5 (1–2.2)	1.6 ± 0.5 (1–2.2)	0.073
PYY (pg/mL)	81.5 ± 17.5 (59.9–118.4)	82.8 ± 22.8 (52.8–116.6)	75.6 ± 23.9 (54.3–113.8)	0.709
α-MSH (pg/mL)	13.9 ± 2.1 (10.4–17.1)	13.1 ± 1.8 (10.3–15.1)	14.6 ± 2.4 (12.4–17.2)	0.317

* Data are presented as mean ± SD (range); ASP: Agouti signaling protein; NPY: Neuropeptide Y; POMC: Proopiomelanocortin; KISS: Kisspeptin; PYY: Putative protein Y; α-MSH: α-Melanocyte-stimulating hormone.

**Table 3:** Levels of hormones depending to the type of bacterial infection *.

	*P. aeruginosa* 5 F/4 M	*S. aureus* 4 F/7 M	Coinfected with *P. aeruginosa* and *S. aures* 4 F/5 M	Uninfected 4 F/5 M	*p*
Ghrelin (pg/mL)	605 ± 242.8 (192.3–828)	634.3 ± 147.2 (435.1–1070.5)	616.8 ± 240 (238.3–1013.8)	829.7 ± 124.9 (682.3–1070.5)	0.054
ASP (pg/mL)	14.9 ± 2.6 (11.9–18.7)	14.4 ± 2.1 (12–17.9)	13.9 ± 2.61 (11.7–19.1)	13.4 ± 1.6 (12–16.5)	0.349
NPY (pg/mL)	290.9 ± 63.4 (210.6–376.4)	291.8 ± 44.4 (220.6–381.6)	282 ± 60.7 (211.1–381.6)	261.7 ± 61.2 (167.6–377.1)	0.349
POMC (ng/mL)	5 ± 3.2 (2.1–10.9)	5 ± 3.9 (2–15.5)	6.7 ± 4.9 (1.9–14.6)	8 ± 5 (2–15.5)	0.349
KISS (ng/mL)	1.4 ± 0.3 (1–2.1)	1.8 ± 0.4 (1.1–3)	1.7 ± 0.6 (1.1–3)	1.9 ± 0.4 (1.2–2.5)	0.063
PYY (pg/mL)	72.6 ± 20.5 (54.3–116.6)	82.7 ± 16.6 (60.7–118.4)	74.2 ± 16.7 (52.8–101.6)	89.2 ± 21 (61.3–118.4)	0.437
α-MSH (pg/mL)	13 ± 2 (10.3–17.2)	13.7 ± 1.8 (10.4–16.6)	13.8 ± 2.5 (11–17.1)	14.2 ± 2.3 (11.4–17.1)	0.996

* Data are presented as mean ± SD (range); ASP: Agouti signaling protein; NPY: Neuropeptide Y; POMC: Proopiomelanocortin; KISS: Kisspeptin; PYY: Putative protein Y; α-MSH—α-Melanocyte-stimulating hormone.

## Data Availability

The datasets generated during and/or analyzed during the current study are not publicly available but are available from the corresponding author on reasonable request.

## References

[1] Schluchter M.D., Konstan M.W., Drumm M.L., Yankaskas J.R., Knowles M.R. (2006). Classifying Severity of Cystic Fibrosis Lung Disease Using Longitudinal Pulmonary Function Data. Am. J. Respir. Crit. Care Med..

[2] Szczesniak R., Heltshe S.L., Stanojevic S., Mayer-Hamblett N. (2017). Use of FEV1 in Cystic Fibrosis Epidemiologic Studies and Clinical Trials: A Statistical Perspective for the Clinical Researcher. J. Cyst. Fibros..

[3] Galiniak S., Mołoń M., Rachel M. (2022). Links between Disease Severity, Bacterial Infections and Oxidative Stress in Cystic Fibrosis. Antioxidants.

[4] Kerem E., Viviani L., Zolin A., MacNeill S., Hatziagorou E., Ellemunter H., Drevinek P., Gulmans V., Krivec U., Olesen H. (2014). Factors Associated with FEV1 Decline in Cystic Fibrosis: Analysis of the ECFS Patient Registry. Eur. Respir. J..

[5] Taylor-Robinson D., Whitehead M., Diderichsen F., Olesen H.V., Pressler T., Smyth R.L., Diggle P. (2012). Understanding the Natural Progression in %FEV1 Decline in Patients with Cystic Fibrosis: A Longitudinal Study. Thorax.

[6] Earnest A., Salimi F., Wainwright C.E., Bell S.C., Ruseckaite R., Ranger T., Kotsimbos T., Ahern S. (2020). Lung Function over the Life Course of Paediatric and Adult Patients with Cystic Fibrosis from a Large Multi-Centre Registry. Sci. Rep..

[7] Coutinho H.D.M., Falcão-Silva V.S., Gonçalves G.F. (2008). Pulmonary Bacterial Pathogens in Cystic Fibrosis Patients and Antibiotic Therapy: A Tool for the Health Workers. Int. Arch. Med..

[8] Chiappini E., Taccetti G., de Martino M. (2014). Bacterial Lung Infections in Cystic Fibrosis Patients: An Update. Pediatr. Infect. Dis. J..

[9] Patient Registry|Cystic Fibrosis Foundation. https://www.cff.org/medical-professionals/patient-registry.

[10] Bhagirath A.Y., Li Y., Somayajula D., Dadashi M., Badr S., Duan K. (2016). Cystic Fibrosis Lung Environment and *Pseudomonas aeruginosa* Infection. BMC Pulm. Med..

[11] John A., Goździk-Spychalska J., Durda-Masny M., Czaiński W., Pawłowska N., Wlizło J., Batura-Gabryel H., Szwed A. (2021). *Pseudomonas aeruginosa*, the Type of Mutation, Lung Function, and Nutritional Status in Adults with Cystic Fibrosis. Nutrition.

[12] Durda-Masny M., Goździk-Spychalska J., John A., Czaiński W., Stróżewska W., Pawłowska N., Wlizło J., Batura-Gabryel H., Szwed A. (2021). The Determinants of Survival among Adults with Cystic Fibrosis—A Cohort Study. J. Physiol. Anthropol..

[13] Galiniak S., Podgórski R., Rachel M., Mazur A. (2022). Serum Leptin and Neuropeptide Y in Patients with Cystic Fibrosis—A Single Center Study. Front. Med..

[14] Cohen R.I., Tsang D., Koenig S., Wilson D., McCloskey T., Chandra S. (2008). Plasma Ghrelin and Leptin in Adult Cystic Fibrosis Patients. J. Cyst. Fibros..

[15] Galiniak S., Podgórski R., Rachel M., Mazur A. (2022). Serum Levels of Hormones Regulating Appetite in Patients with Cystic Fibrosis—A Single-Center, Cross-Sectional Study. Front. Endocrinol..

[16] Beck B. (2006). Neuropeptide Y in Normal Eating and in Genetic and Dietary-Induced Obesity. Philos. Trans. R. Soc. B Lond. Biol. Sci..

[17] Liu Y., Albrecht E., Schering L., Kuehn C., Yang R., Zhao Z., Maak S. (2018). Agouti Signaling Protein and Its Receptors as Potential Molecular Markers for Intramuscular and Body Fat Deposition in Cattle. Front. Physiol..

[18] Voisey J., van Daal A. (2002). Agouti: From Mouse to Man, from Skin to Fat. Pigment Cell Res..

[19] Vehapoğlu A., Türkmen S., Terzioğlu Ş. (2016). Alpha-Melanocyte-Stimulating Hormone and Agouti-Related Protein: Do They Play a Role in Appetite Regulation in Childhood Obesity?. J. Clin. Res. Pediatr. Endocrinol..

[20] Podgórski R., Galiniak S., Mazur A., Domin A. (2023). The Association of the Hypothalamic-Pituitary-Adrenal Axis with Appetite Regulation in Children with Fetal Alcohol Spectrum Disorders (FASDs). Nutrients.

[21] Millington G.W. (2007). The Role of Proopiomelanocortin (POMC) Neurones in Feeding Behaviour. Nutr. Metab..

[22] Böhm M., Grässel S. (2012). Role of Proopiomelanocortin-Derived Peptides and Their Receptors in the Osteoarticular System: From Basic to Translational Research. Endocr. Rev..

[23] Dudek M., Ziarniak K., Sliwowska J.H. (2018). Kisspeptin and Metabolism: The Brain and Beyond. Front. Endocrinol..

[24] Cahill F., Ji Y., Wadden D., Amini P., Randell E., Vasdev S., Gulliver W., Sun G. (2014). The Association of Serum Total Peptide YY (PYY) with Obesity and Body Fat Measures in the CODING Study. PLoS ONE.

[25] Graham B.L., Steenbruggen I., Miller M.R., Barjaktarevic I.Z., Cooper B.G., Hall G.L., Hallstrand T.S., Kaminsky D.A., McCarthy K., McCormack M.C. (2019). Standardization of Spirometry 2019 Update. An Official American Thoracic Society and European Respiratory Society Technical Statement. Am. J. Respir. Crit. Care Med..

[26] Lee T.W.R., Brownlee K.G., Conway S.P., Denton M., Littlewood J.M. (2003). Evaluation of a New Definition for Chronic *Pseudomonas aeruginosa* Infection in Cystic Fibrosis Patients. J. Cyst. Fibros..

[27] Weinstein M.P., Patel J.B. (2018). Methods for Dilution Antimicrobial Susceptibility Tests for Bacteria That Grow Aerobically: M07-A11.

[28] Soltman S., Hicks R.A., Naz Khan F., Kelly A. (2021). Body Composition in Individuals with Cystic Fibrosis. J. Clin. Transl. Endocrinol..

[29] Nagy R., Gede N., Ocskay K., Dobai B.-M., Abada A., Vereczkei Z., Pázmány P., Kató D., Hegyi P., Párniczky A. (2022). Association of Body Mass Index With Clinical Outcomes in Patients With Cystic Fibrosis: A Systematic Review and Meta-Analysis. JAMA Netw. Open.

[30] Sheikh S., Zemel B.S., Stallings V.A., Rubenstein R.C., Kelly A. (2014). Body Composition and Pulmonary Function in Cystic Fibrosis. Front. Pediatr..

[31] Harindhanavudhi T., Wang Q., Dunitz J., Moran A., Moheet A. (2020). Prevalence and Factors Associated with Overweight and Obesity in Adults with Cystic Fibrosis: A Single-Center Analysis. J. Cyst. Fibros..

[32] Monajemzadeh M., Mokhtari S., Motamed F., Shams S., Ashtiani M.T.H., Abbasi A., Sani M.N., Sadrian E. (2013). Plasma Ghrelin Levels in Children with Cystic Fibrosis and Healthy Children. Arch. Med. Sci..

[33] Nowak J.K., Szczepanik M., Trypuć M., Pogorzelski A., Bobkowski W., Grytczuk M., Minarowska A., Wójciak R., Walkowiak J. (2020). Circulating Brain-Derived Neurotrophic Factor, Leptin, Neuropeptide Y, and Their Clinical Correlates in Cystic Fibrosis: A Cross-Sectional Study. Arch. Med. Sci..

[34] Galiniak S., Podgórski R., Rachel M., Mazur A. (2022). Serum Kisspeptin and Proopiomelanocortin in Cystic Fibrosis: A Single Study. Sci. Rep..

[35] Qi H., Liu H., Zheng P., He J. (2023). Lack of association between leptin concentrations and cystic fibrosis: A meta-analysis and regression. Front. Endocrinol..

[36] Blackman S.M., Tangpricha V. (2016). Endocrine Disorders in Cystic Fibrosis. Pediatr. Clin. N. Am..

[37] Rachel M., Topolewicz S., Śliwczyński A., Galiniak S. (2020). Managing Cystic Fibrosis in Polish Healthcare. Int. J. Environ. Res. Public Health.

[38] Ahmed M., Ong K., Thomson A., Dunger D. (2004). Reduced Gains in Fat and Fat-Free Mass, and Elevated Leptin Levels in Children and Adolescents with Cystic Fibrosis. Acta Paediatr..

[39] Liang R., Zhang W., Song Y.-M. (2013). Levels of Leptin and IL-6 in Lungs and Blood Are Associated with the Severity of Chronic Obstructive Pulmonary Disease in Patients and Rat Models. Mol. Med. Rep..

[40] Mitri C., Xu Z., Bardin P., Corvol H., Touqui L., Tabary O. (2020). Novel Anti-Inflammatory Approaches for Cystic Fibrosis Lung Disease: Identification of Molecular Targets and Design of Innovative Therapies. Front. Pharmacol..

[41] Finck B.N., Johnson R.W. (2000). Tumor Necrosis Factor (TNF)-Alpha Induces Leptin Production through the P55 TNF Receptor. Am. J. Physiol. Regul. Integr. Comp. Physiol..

[42] Malhotra S., Hayes D., Wozniak D.J. (2019). Cystic Fibrosis and *Pseudomonas aeruginosa*: The Host-Microbe Interface. Clin. Microbiol. Rev..

[43] Stelzer I., Zelzer S., Raggam R.B., Prüller F., Truschnig-Wilders M., Meinitzer A., Schnedl W.J., Horejsi R., Möller R., Weghuber D. (2012). Link between Leptin and Interleukin-6 Levels in the Initial Phase of Obesity Related Inflammation. Transl. Res..

[44] Pîrsean C., Neguț C., Staden R.-I.S., Dinu-Pirvu C.E., Armean P., Udeanu D.I. (2019). The Salivary Levels of Leptin and Interleukin-6 as Potential Inflammatory Markers in Children Obesity. PLoS ONE.

[45] Chiu C.Z., Wang B.W., Shyu K.G. (2015). Molecular regulation of the expression of leptin by hypoxia in human coronary artery smooth muscle cells. J. Biomed. Sci..

[46] Grosfeld A., Zilberfarb V., Turban S., André J., Guerre-Millo M., Issad T. (2002). Hypoxia increases leptin expression in human PAZ6 adipose cells. Diabetologia.

[47] Urquhart D.S., Montgomery H., Jaffé A. (2005). Assessment of hypoxia in children with cystic fibrosis. Arch. Dis. Child..

[48] Polito R., Nigro E., Elce A., Monaco M.L., Iacotucci P., Carnovale V., Comegna M., Gelzo M., Zarrilli F., Corso G. (2019). Adiponectin Expression Is Modulated by Long-Term Physical Activity in Adult Patients Affected by Cystic Fibrosis. Mediat. Inflamm..

[49] Bouloumie A., Marumo T., Lafontan M., Busse R. (1999). Leptin Induces Oxidative Stress in Human Endothelial Cells. FASEB J..

[50] Schroyen B., Guimarães E.L., Dollé L., Coulon S., Empsen C., Nyssen M., Geerts A., Colle I., Geerts A., van Grunsven L.A. (2012). Leptin-Mediated Reactive Oxygen Species Production Does Not Significantly Affect Primary Mouse Hepatocyte Functions in Vitro. Eur. J. Gastroenterol. Hepatol..

[51] Berger S., Polotsky V.Y. (2018). Leptin and Leptin Resistance in the Pathogenesis of Obstructive Sleep Apnea: A Possible Link to Oxidative Stress and Cardiovascular Complications. Oxid. Med. Cell. Longev..

[52] Moliteo E., Sciacca M., Palmeri A., Papale M., Manti S., Parisi G.F., Leonardi S. (2022). Cystic Fibrosis and Oxidative Stress: The Role of CFTR. Molecules.

[53] Wang B., Wood I.S., Trayhurn P. (2008). Hypoxia Induces Leptin Gene Expression and Secretion in Human Preadipocytes: Differential Effects of Hypoxia on Adipokine Expression by Preadipocytes. J. Endocrinol..

[54] Cohen R.I., Chandra S., Koenig S., Tsang D., Wilson D., McCloskey T. (2010). Ghrelin Receptor Expression in Lymphocytes Isolated from Adult Cystic Fibrosis Patients. Respiration.

